# HIV-1 gp120 N-linked glycosylation differs between plasma and leukocyte compartments

**DOI:** 10.1186/1743-422X-5-14

**Published:** 2008-01-23

**Authors:** Yung Shwen Ho, Ana B Abecasis, Kristof Theys, Koen Deforche, Dominic E Dwyer, Michael Charleston, Anne Mieke Vandamme, Nitin K Saksena

**Affiliations:** 1 Retroviral Genetics Laboratory, Centre for Virus Research, Westmead Millennium Institute, Westmead Hospital, University of Sydney, Westmead NSW. 2145 Sydney. Australia; 2 Clinical and Epidemiological Virology, Rega Institute for Medical Research, Leuven, Belgium; 3 Centre for Infectious Diseases and Microbiology Laboratory Services, ICPMR, Westmead Hospital, Westmead NSW 2145, Australia; 4 School of Information Technologies, University of Sydney, Camperdown NSW 2006, Australia

## Abstract

**Background:**

N-linked glycosylation is a major mechanism for minimizing virus neutralizing antibody response and is present on the Human Immunodeficiency Virus (HIV) envelope glycoprotein. Although it is known that glycosylation changes can dramatically influence virus recognition by the host antibody, the actual contribution of compartmental differences in N-linked glycosylation patterns remains unclear.

**Methodology and Principal Findings:**

We amplified the *env *gp120 C2-V5 region and analyzed 305 clones derived from plasma and other compartments from 15 HIV-1 patients. Bioinformatics and Bayesian network analyses were used to examine N-linked glycosylation differences between compartments. We found evidence for cellspecific single amino acid changes particular to monocytes, and significant variation was found in the total number of N-linked glycosylation sites between patients. Further, significant differences in the number of glycosylation sites were observed between plasma and cellular compartments. Bayesian network analyses showed an interdependency between N-linked glycosylation sites found in our study, which may have immense functional relevance.

**Conclusion:**

Our analyses have identified single cell/compartment-specific amino acid changes and differences in N-linked glycosylation patterns between plasma and diverse blood leukocytes. Bayesian network analyses showed associations inferring alternative glycosylation pathways. We believe that these studies will provide crucial insights into the host immune response and its ability in controlling HIV replication *in vivo*. These findings could also have relevance in shielding and evasion of HIV-1 from neutralizing antibodies.

## Introduction

The HIV-1 envelope (*env*) gp120 region plays a crucial role in the entry of HIV-1 into target cells through the fusion of viral envelope with the target cell membrane. Variable regions (V1-V5) in *env *are spaced between the conserved regions (C1-C5). Both N-linked and O-linked glycans are present on the HIV envelope glycoprotein. O-linked glycans are present on several unidentified serine or threonine residues in *env *gp120, but very little is known about their actual role in governing the viral phenotype of both HIV and simian immunodeficiency virus (SIV) [1,2]. In contrast, N-linked glycans comprise about 50% of the mass of the *env *gp160 [3]. These sugar moieties are involved in various activities such as metabolism, transport, structural maintenance of the cell and protein, protein folding, recognition of particular cell types and adhesion to other cells. The N-linked glycosylation (NLG) of viral envelope proteins, through the formation of a "glycan shield", is one of the major mechanisms for blocking or minimizing virus neutralizing antibody response [4]. which promotes viral persistence and immune evasion. This has been demonstrated in SIV [5,6], HIV-1 [4,7] influenza virus [8], hepatitis B virus [9] and the Lactate Dehydrogenase-elevating Virus [10]. Despite considerable genetic variation in HIV strains, the number of NLG are often found to be around 25 sites in the HIV-1 *env *gp120 region [11], suggesting that strong selective pressures maintain this number [4]. The HIV envelope "glycan shield" is known to evolve in response to host antibodies [4] and it is thought that the density of gp120 NLG is a significant obstacle to the design of effective vaccine and elicitation of humoral immune responses. Any alteration or positional shift of a glycosylation site (commonly seen in HIV and SIV glycoproteins) can have dramatic consequences for the virus and its recognition by the antibody.

Although recent studies have shown compartmentalization of HIV-1 NLG sites between viral populations in plasma and the female genital tract [12,13], the critical issue of possible differences in NLG of HIV-1 strains derived from cell-associated and cell-free compartments remains unexplored. Such differences are important to future drug development because the drugs used in highly active antiretroviral therapy (HAART) primarily target plasma or cell-free virus. Cell-free virus has a high turnover rate (< 6 hours) [4] and therefore has a strong need to maintain viral integrity through constant shielding from host antibodies. In contrast, cell-associated virus are kept away from neutralizing antibodies and can remain integrated in the human genome indefinitely. They can produce viral progeny upon activation *in vivo *and this acts as an impediment to the success of therapy. The integrated provirus concealed in diverse blood leukocyte populations is one strategy HIV uses to avoid immune detection. Given the incessant virus trafficking between cellfree and cell-associated compartments, a clear determination of differences of HIV populations in plasma and diverse cell types is needed to understand critical molecular determinants for viral survival, turnover, evasion and adaptation *in vivo*. The relevance of NLG is also known for many other viruses [14-16]. Together, these studies imply that the virus-producing cell type is an important factor, which may be crucial in viral tropism and transmissibility *in vivo*.

The role of single amino acid residue changes in the HIV-1 *env *in its adaptation to cellular compartments remains similarly unexplored. Given that different cellular compartments have different immune functions in our body, we suspect that the virus populations within them are subjected to distinct selection pressures, as opposed to freely circulating virus in plasma [17]. These distinct selective forces may further exert influence on the make-up of NLG, depending on the cell type. This evolutionary make-up may, in turn, define biological and functional aspects of viral variants in a given environment. Different glycosylation sites have been shown to offer variable sensitivity to antibody-mediated neutralization [4], such as sites in the *env *hypervariable C3 and C5 regions. The sites around the base of the V3 loop have been consistently found to be associated with neutralization sensitivity, especially in HIV-1 subtype B viruses [4]. As the majority (17 of 25) of NLG sites are concentrated in the *env *C2-V5 region of gp120, and given the functional relevance of glycans in HIV pathogenesis, we chose this region for the study of HIV-1 glycosylation patterns in cell-free and cell-associated compartments.

We analyzed 305 clones of HIV-1 variants derived from plasma and diverse blood leukocytes (whole Peripheral Blood Mononuclear Cell (PBMC), CD4^+ ^T cells, CD8^+ ^T cells and monocytes) from 15 HIV-1 infected patients on HAART, each displaying different levels of plasma viremia and T cell counts. In addition to the patient specific changes observed in our analyses, we further found evidence in favor of compartmental NLG differences and distinct cell-specific molecular changes in the *env *C2-V3 region of HIV-1.

## Results

### Phylogenetic analysis

**Figure 1:**
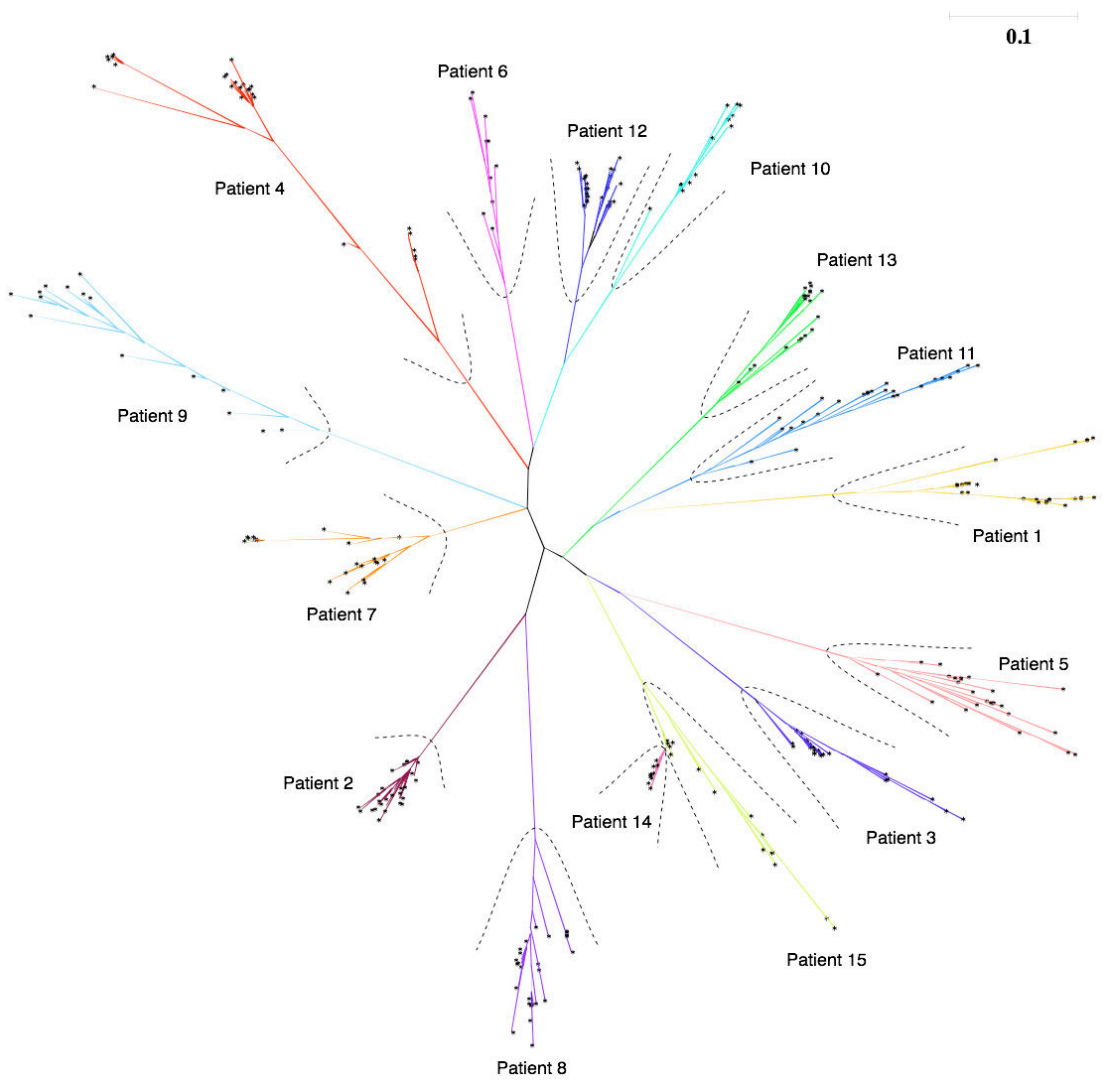
**Phylogenetic tree analysis showing patient sequence purity**. Phylogenetic analysis on the 305 HIV-1 *env *gp120 C2-V5 region sequences from plasma, peripheral blood mononuclear cells, CD4^+ ^T cells, CD8^+ ^T cells and monocytes. Using the ProML program of the PHYLIP software package, a maximum likelihood phylogenetic tree was calculated for our patient sequences. The branch lengths are scaled to distance. A single asterisk represents each sequence from our dataset. Individual sequences are not identified as it is only the broad pattern of clustering that is of interest here. Distinct clustering of patient-related sequences can be seen from the phylogenetic tree.

Phylogenetic tree reconstruction using a maximum likelihood heuristic search algorithm showed patient-specific clustering of virus from all compartments, confirming the patient origin of HIV-1 variants and the absence of cross-patient contamination. Further, within each patient there was distinct clustering of HIV-1 sequences from each compartment, confirming the purity of diverse blood leukocytes and plasma samples (Figure 1). This provided us with a platform to compare our data both at the level of single amino acid mutations and NLG differences across cell-free and cellassociated compartments.

### Signature pattern analysis

From our signature pattern analysis, we found seven single amino acid differences from the 305 HIV-1 sequences across five different compartments (Table 1). These positions are referenced to the HIV-1 HXB2 prototype using the referencing guidelines available from the Los Alamos HIV sequence database website [18]. The columns in Table 1 categorize our data into plasma and blood leukocyte compartments, while the rows in Table 1 represent the amino acid differences at each of the identified sites. As shown in Table 1, CD4^+ ^T cells, CD8^+ ^T cells and monocyte-derived sequences were found with the amino acid asparagine (N) at position 279, whereas the PBMC and plasma-derived sequences were found to have aspartic acid (D) at the same position. The amino acid lysine (K) was uniquely seen in monocyte-derived HIV sequences at positions 335 and 350, whereas other compartments showed arginine (R) at that position. Further amino acid differences across compartments were found at positions 320, 336, 360 and 440 as illustrated in Table 1. Statistical analysis confirmed a significant association (p = 0.044) in the observed single amino acid differences between CD4^+ ^T cell and plasma-derived sequences at position 360. However a lost of significance was found (p = 0.22) when we correct for multiple comparisons.

**Table 1:** Single amino acid differences found across plasma and diverse blood leukocyte population *in vivo*.

HXB2	CD4^+^	CD8^+^	Monocytes	PBMC	Plasma
279	Asparagine (N)			Aspartic acid (D)	
336	Threonine (T)			Alanine (A)	
335	Arginine (R)		Lysine (K)	Arginine (R)	
350	Arginine (R)		Lysine (K)	Arginine (R)	
320	Alanine (A)	Threonine (T)			Alanine (A)
440	Arginine (R)		Serine (S)		Arginine (R)
360	Isoleucine (I)		Alanine	Valine(v)	Alanine (A)

Each column represents a particular compartment from which sequences were derived. Distinct signature pattern differences are shown in the rows with the corresponding amino acid variations. The locations of these variations are aligned with those of the HIV-1 HXB2 reference strain using the guidelines available on the HIV Database website, Los Alamos, NM, USA [18].

### N-linked glycosylation analysis

**Figure 2:**
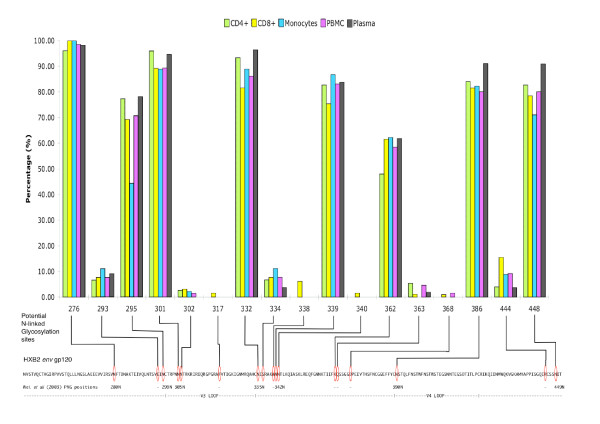
**N-linked glycosylation frequency observed in the *env *gp120 C2-V5 region across plasma and cellular viral sequences**. Frequency of HIV-1 N-linked glycosylation sites in plasma and diverse blood leukocyte populations. The X-axis represents the potential N-linked glycosylation sites identified in our study. The Y-axis shows the percentage frequency of the sequences for each compartment found with NLG at the relevant position. Below the bar chart is the *env *gp120 sequence of our reference HIV-1 HXB2 strain. Lines from the X-axis to the reference sequence illustrate where the observed NLG in our data would be on HXB2. NLG sites observed were matched with those reported by Wei *et al *[4] using the GenBank sequence U21135. The numbers below each red oval indicates where you might associate our NLG site with those from the study by Wei *et al *[4] in the GenBank sequence U21135. Positions with no visual correlation are indicated with a dash '-'.

N-linked glycosylation analysis using the N-Glycosite program [11] identified 17 NLG sites from our 305 HIV-1 *env *gp120 gap-stripped protein sequences (Figure 2). The positions of the sites are referenced using the HXB2 prototype sequence as described above. On the whole, the NLG frequencies were found to vary greatly from site to site. Positions 276, 295, 301, 332, 339, 386 and 448 had a high frequency of ≥70% in our sequences. Positions 293, 302, 317, 334, 338, 340, 363, 368 and 444 had a low frequency (<16%) in our sequence population. The number of glycosylation sites varied significantly between patients (p < 2.2 × 10^-16^) in our inter-patient analysis. Among the NLG sites identified, the one at position 338 from patient 13 was unique to the HIV-1 strain and had not previously been recorded in the Los Alamos HIV database.

### Empirical statistical analysis

The χ^2 ^test for the comparison of frequencies across five different compartment using a 5 × 2 contingency table gave a p-value of 0.0015 for position 295. Fisher's exact test for the comparison of frequencies observed from plasma versus all cell-types (CD4, CD8, Monocytes, PBMC) showed significant difference (p = 0.04) in the frequencies observed at position 448. We are aware that these values might be affected by our treatment of several clones from the same patient and from the same compartment, as the different sequences are not truly independent events. Hence, we extended this analysis further to include only the median number of NLG sites per patient per compartment and not each individual count of NLG sites per sequence. While this procedure eliminates the effect of non-independence, it also lessens the number of observations and possibly the statistical power. When we analysed the median number of NLG sites between compartments with the gap-stripped sequences (Tables 2), the Kruskal-Wallis test confirmed a significantly higher median number of NLG sites observed in plasma than in cellular compartments (p = 0.022) when sequences from PBMC, CD4^+ ^T cells, CD8^+ ^T cells, monocytes were grouped together. Moreover, this difference was slightly more pronounced when we compared plasma and monocyte sequences (p = 0.017; Table 2). Repeating the statistical analysis with Bonferroni correction for multiple comparisons gave us a p-value of 0.114 and 0.085 respectively. No statistical differences in the median number of N-linked glycosylation sites were found in the gap-inclusive alignment.

**Table 2:** Statistical comparison on the number of glycosylation sites between compartments using the gap-stripped sequences.

Number of NLG sites	CD4^+^	CD8^+^	Monocytes	PBMC	Plasma
CD4^+^	-	0.8713	0.1711	0.7645	0.08367
CD8^+^	-	-	0.2908	0.9132	0.0962
Monocytes	-	-	-	0.3412	**0.01696**
PBMC	-	-	-	-	0.0818
Grouped cells	-	-	-	-	**0.02281**

Above are the p-values resulting from the Kruskal-Wallis test when we compared for statistical differences in the observed number of glycosylation sites across different compartments using the gap-stripped sequences. The category "Grouped cells" represents sequences from cellular compartments (CD4^+^, CD8^+ ^T cells, Monocytes and PBMC). The gap-inclusive results are not shown as no statistical significant difference was observed (see text).

### Bayesian network analysis

**Figure 3:**
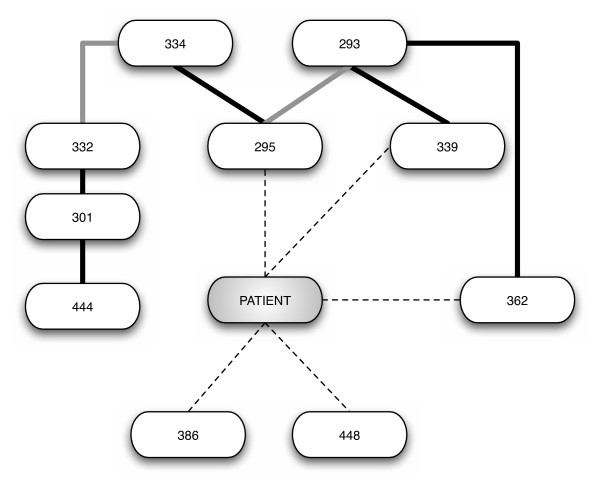
**Bayesian network associations found between observed N-linked glycosylation sites**. Representation of the Bayesian network analysis results, generated as described in the methods section. Only arcs with a bootstrap support of at least 70% are presented. Associations between patient ID and glycosylation sites are dashed. Associations between glycosylation sites that can be structurally explained are colored grey. Associations between glycosylation sites that cannot be structurally explained are colored black.

We found a strong statistical significance between patient-specific sequences and the presence/absence of certain glycosylation sites at positions 295, 339, 362, 386 and 448 (Figure 3). This analysis also showed an interesting dependency between NLG sites at different positions, shown in Figure 3. While some of those dependencies are expected from N-linked glycosylation motif (like 293–295 and 332–334), the associations found between other sites 301–444, 293–362, 295–334, and 301–332 respectively, are novel.

To deduce the possible biological and functional relevance of these findings, we also compared the 17 potential NLG sites found in our study to functionally analyze N-linked glycosylation sites published by Wei *et al *[4]. Most of the sites noted in our study matched the N-linked glycosylation sites (from GenBank U21135) of functional relevance described by Wei *et al *[4] (Figure 2). Due to sequence variability, some glycosylation sites were very close to each other, we have chosen to associate more than one site to the NLG positions reported by Wei *et al *[4]. For example, positions 301 and 302, being 1 amino acid away from one another, are likely to represent NLG 305N from their study [4]. We took the same view for position pair 332 and 334, corresponding to NLG 335N, and position pair 339 and 340, corresponding to NLG 342N from Wei *et al *[4].

Searching for the best Bayesian network representation for this dataset is an extremely difficult task considering our relatively small sample size against the number of variables (potential NLG sites) we have account for. This pitfall was overcome by combining the Bayesian network analysis with a bootstrap approach. The strength of the arcs is proportional to its bootstrap support and not to the the importance of the conditional independencies to the joint probability of the network. Overall, these analyses have allowed us to obtain a detailed profile of potential N-linked glycosylation distribution and possible inter-relationship between NLG sites in HIV-1 *env *gp120 sequences between different blood leukocyte and plasma-derived HIV-1 populations *in vivo*.

## Discussion

In order for HIV to be successful in evading the immune system, both cell-free and cell-associated forms of HIV must adopt distinct molecular strategies to adapt and survive *in vivo*. Such adaptation includes the modulation of N-linked glycosylation and cell-specific single amino acid changes in HIV. Given the importance of the envelope glycoprotein in neutralization, pathogenesis, tropism and viral evasion, we analysed the C2-V5 region of 305 HIV-1 *env *gp120 sequences from plasma and diverse blood leukocytes of 15 patients on HAART. Single amino acid differences specific to plasma and cellular compartments were observed. Notable was the presence of lysine (K) in monocytes at positions 335 and 350 whereas these positions have arginine (R) in the other compartments. This suggests that positions 335 and 350 have the greatest need to maintain a basic amino acid residue (lysine) in monocytes, compared with the other compartments. This difference may have a role in viral adaptation to monocytes and possibly relevant to monocyte tropism. In addition, the isoleucine at position 360 was unique to CD4^+ ^T cells (p < 0.044). Additionally the presence of valine was unique to PBMC (and absent in plasma and the three other cell types) at position 360. Although the monocyte compartment is a subset of the PBMC compartment, the majority of PBMC sequences were found with valine (V) and the monocyte sequences with alanine (A). We believe that although all the cellular compartments we analyzed were derived from the whole PBMC, the valine may be specific to a leukocyte subset, which was not analyzed in this study, due to the limitation of human bleed obtained from each patient. Nonetheless, this difference between monocytes and PBMC supports our observation that compartmental-specific amino acid changes in HIV-1 *env *gp120 are present. Our results agree with previous work on single amino acid changes in the *env *gene in association with viral tropism and pathogenicity [19]. A recent study by Clevestig *et al *[20], shows the V3 loop glycan (especially the sequon motif NNT) to be critical for CCR5 use, which may have a direct role in HIV tropism, further supporting our conclusions. We believe that these single amino acid differences could be vital to HIV and are acquired through intra-host evolution in order to successfully adapt and thrive in different *in vivo *environments.

The modulation of N-linked glycosylation sites in the HIV-1 envelope has been known to facilitate viral evasion from the host immune system. Similar to the signature pattern analysis, the N-linked glycosylation analysis yields interesting results showing compartmental variations and possible associations among glycosylation sites. An examination of the NLG frequencies along the C2-V5 region (Figure 2) revealed that plasma virus had a similar or higher percentage of its sequences glycosylated at the identified sites, compared to the other compartmental viruses. A possible explanation for this is that the differences in N-linked glycosylation sites in plasma may be required for the maintenance of infectious potential. Through the suppression of HIV-1 in CD4^+ ^T cells and plasma in our HAART patient pool, glycosylation change in the virus may enable it to overcome the selection pressures from the antiretroviral regimen. Further examination of the NLG sites using Bayesian networks found unique associations between sites 301–444, 293–362, 295–334, and 301–332. These associations could indicate alternative pathways for glycosylation and possible simultaneous co-selection of glycosylation sites. Similar to the study of evolutionary interactions between NLG sites by Poon Art F, Y [21], we believe that these evolutionary mechanisms is important to HIV for its struggle against host immune selection pressure. Since all our work was performed directly on *ex vivo*-collected cells from each patient, our results reflect the closest possible snapshot to the *in vivo *situation.

We examined the variation in the number of glycosylation sites among compartments (gap-free and gap-inclusive) to infer a global picture of HIV-1 *in vivo*. The trade-off between them is an important issue to consider in this analysis. While using the gap-inclusive alignment might include unavoidable interference due to the uncertainty of the alignment in the hypervariable region of *env*, the use of the gap-stripped alignment might also introduce an oversight of the observed glycosylation due to the potential removal of important glycosylation sites from the alignment. Therefore, we consider both analyses in the results section. The results were twofold: no statistical significance in the total number of glycosylation sites between plasma and different cellular compartment sequences was found using the gap-inclusive alignment. However when we performed the gap-stripped analysis, we observed a significant difference (p = 0.022) between plasma and cellular compartment sequences. This is consistent with the notion that the number of glycosylation sites is highly conserved in HIV-1 [4]. The disparity between these results suggests that there is a difference in the number of glycosylation sites between plasma virus and cellular virus in the less conserved regions of HIV-1 *env*. Despite the loss of statistical significance when we applied the Bonferroni correction for multiple comparison from P = 0.022 to P = 0.115, we believe that it is still an important finding together with the rest of our statical analysis. This is because by correcting for multiple comparisons, we are also increasing the risk of making a type II error which might lead us to not report a correlation when there is one. Between both gap-stripped and gap-inclusive analysis, the gap-inclusive alignment gives us the actual number of glycosylation sites, as no glycosylation sites were omitted in the evaluation. Not finding any significant differences in this gap-inclusive alignment indicates that possible selective pressure to remove glycosylation sites from the more conserved part of *env *was compensated by the creation of new glycosylation sites in those parts of *env *that better tolerate substitutions, insertions and deletions.

Even though we found evidence for compartmentalization of HIV-1 across plasma and diverse blood leukocyte populations *in vivo*, it is important to note that the NLG observed in our sequences are distinctly patient-specific (p ≤ 2.2 × 10^-16^). This supports our belief that the selection of NLG sites are primarily dependent on the patient's immune response. The choice to examine 305 sequences from 15 independent patients with a range of CD4^+ ^T cell counts and plasma viral load in our study has allowed us to infer a balanced macroscopic perspective of HIV-1 adaptation *in vivo *during therapy. These analyses are the first to provide a detailed comparison of cell-free and cellassociated virus, especially in individual leukocyte types. Previous studies by Hanna *et al *[14] and Lin *et al *[15] support the validity of our studies on diverse cell types and add further credence to the functional basis of different NLG frequencies in plasma and cell-associated virus. Together, these findings might provide a new direction and perspective into the role of glycosylation in diverse compartments *in vivo *and these data may have relevance in HIV immune recognition, viral adaptation, vaccine strategies and HIV pathogenesis in general.

## Conclusion

This study examined single cell/compartment-specific amino acid variations and unique differences in N-linked glycosylation patterns between plasma and diverse blood leukocytes. It has provided deeper insights into how HIV may evade antibodies and maintain its pathogenic potential. Bayesian analysis has shown associations that suggest possible glycosylation pathways. We believe that these analyses provide useful insights into the host immune response and its ability in controlling HIV replication *in vivo*. Further, this enhance our understanding of pathogenous differences between cell-free and cell-associated HIV-1. Consequently, these analyses will allow further biological and functional assessment of such molecular changes in the context of viral escape, adaptation and reservoir establishment *in vivo*. A better understanding of diverse N-linked glycosylation sites and their functional role may provide useful strategies for choosing and eliminating the "right" N-linked glycosylation site(s), thus facilitating the design of more effective envelope-based immunogens that elicit broad neutralization antibody responses.

## Methods

### Consent

This work is carried out in accordance with the human ethics guidelines and scientific principles set out by the National Health and Medical Research Council of Australia (NHMRC). All patients have given written consent on the study and understand that the study will be conducted in a manner conforming to the ethical and scientific principle set out by the NHMRC.

### Patient selection

Fifteen HIV-1 infected patients receiving HAART from the Westmead Hospital in Sydney, Australia, were enrolled in this study after prior consent. These patients exhibited varying plasma viral loads and T cell counts as shown in Table 3. Patients 6, 11, 12, and 13, who were successful in therapy, had plasma viral loads below detectable levels (<50 copies/ml plasma). Patient 9, who was at an advanced stage of therapy, had low viremia. Patients 2, 4, 8, 10, 14 and 15 had varying degrees of plasma viremia after being on HAART for 4 weeks. Patients 1, 3, 5 and 7 had high plasma viral load (>100,000 copies/ml plasma) and were resistant to antiretroviral drugs [22]. No untreated patients could be recruited in this study as every HIV patient in Australia receives treatment for their condition.

**Table 3:** Patients plasma viral load, CD4^+ ^and CD8^+ ^T cell counts.

Patient	CD4^+ ^Count/μl blood	CD8^+ ^Count/μl blood	Plasma Viral Load (RNA c/ml)
1	200	790	> 100,000
2	437	950	5730
3	60	900	> 100,000
4	16	N/A	87700
5	8	172	> 100,000
6	105	675	< 50
7	14	420	> 100,000
8	302	800	60200
9	195	1110	1510
10	135	1647	64500
11	580	870	< 50
12	360	460	< 50
13	1012	2806	< 50
14	180	510	97700
15	340	750	46000

Plasma viral load, CD4^+ ^and CD8^+ ^T cell counts were performed on the samples obtained from 15 patients. The patients showed varied level disease stage according to the T cell numbers and corresponding viral load presented. Patients 1,3,5,7 and 14 were at the late stage of the HIV infection with high plasma viral load (at least 100,000 copies of viral RNA per ml of plasma). Patients 4,8,10 and 15 had intermediate levels of plasma viral load. Patients 2,6,9,11,12 and 13 had low plasma viral load, indicating that they were at a earlier stage of their HIV infection.

### Cell purification and sequence generation

50 ml of blood was collected from each patient. Individual cell types (CD4^+ ^T cells, CD8^+ ^T cells and CD14^+ ^monocytes) were separated from PBMC using magnetic beads coated with monoclonal antibodies (Dynal, Oslo, Norway) using the procedure described and developed by Potter *et al *[17]. FACS analysis of separated cellular fractions showed an average purity of 99.6%. Proviral DNA was extracted from PBMC and individually sorted into cellular fractions using the Qiagen Blood kit (Qiagen, Germany) as per the manufacturer's protocol. A nested polymerase chain reaction (PCR) was used to amplify a 600 bp fragment in the C2-V5 region of *env *gene. Reverse transcription-PCR (RT-PCR) of HIV-1 RNA extracted from plasma using the Qiagen RNA Extraction Kit (Qiagen, Germany) was carried out to amplify viral populations from the cell-free plasma fraction [17]. Independent PCR experiments were performed in triplicate on each separated fraction, and pooled products were used to generate compartment-specific clones (five clones per compartment). To analyse cell-free and cell-associated viral populations from each patient in parallel, the HIV-1 populations from their whole PBMC, CD4^+^, CD8^+ ^T cells, monocytes and plasma were cloned. In each case, the major amplicon population without cloning was first obtained to assess diversity within a patient. Following that, cloning and sequencing were performed as described previously [17] to analyse diversity in each compartment at the quasispecies level, using the major population from the same patient as a comparison. This was performed to derive a clear estimate of intrapatient genetic diversity. Reverse transcription-PCR from plasma was unsuccessful from patient 6, 10 and 13 as these patients had plasma viremia below detectable levels. PCR amplification of CD8^+ ^T cells was not successful for patient 6 and 9; neither was it successful for monocytes for patients 6, 7, 9, 14 and 15 or PBMC for patients 14 and 15. A total of 305 HIV-1 cloned sequences were generated from 15 patients. BLAST searches and phylogenetic analyses (Figure 1) were done to rule out any evidence of laboratory contamination through PCR. Further, manual inspection of all sequences was performed to ensure that the sequenced region was in-frame and there were no significant gene alterations (insertions, deletions and nonsense mutations) in both major populations and clones. All sequences derived from the 15 different patients were identified as subtype B.

All 305 HIV-1 nucleotide sequences from the C2-V5 region of the *env *gp120 region were translated into their protein equivalent using Transeq (EMBOSS) [23]. From the protein sequences, a "gap-inclusive" alignment was created with a multiple sequence alignment program (in this case CLUSTALW [24]) and verified visually. Gaps introduced in sequences by this process correspond to hypothesized insertion or deletion (indel) events, and the alignment is therefore referred to as gap-inclusive. As frequent indels render the alignment very difficult, and in some cases ambiguous, we also created a "gap-stripped" alignment, by removing from the gap-inclusive alignment all sites that contain a gap in any sequence. All alignments mentioned in this paper are considered gap-inclusive unless stated otherwise. The HIV-1 HXB2 envelope sequence was used as our reference.

### Phylogenetic analysis

Phylogenetic reconstructions were performed to confirm the purity of viral sequences at the level of individual patients and each compartment analyzed. Phylogenetic analysis was performed based on the gap-stripped amino acid alignment using maximum likelihood on the ProML program (version 3.66) of the PHYLIP package [25] with the Jones, Taylor and Thornton model of amino acid replacement with a constant rate of change.

### Signature pattern analysis

Signature pattern analysis was performed on the translated protein sequences using the Viral Epidemiology Signature Analysis (VESPA) software [26]. The VESPA software examines single amino acid differences between groups of sequences by creating consensus sequences for each group. In our study, the sequences were grouped into their original host cell types (PBMC, CD4^+ ^T cells, CD8^+ ^T cells, monocytes and plasma) and compared inter-compartmentally. Given the extensive inter-strain/inter-patient variation in our 305 sequences, the majority consensus parameter was used. The "no fixed rates" option was chosen because a fixed rate would not capture the range of diversity observed.

### N-linked glycosylation analysis

N-linked glycosylation analysis was performed on all 305 protein sequences from the C2-V5 region of the *env *gp120 protein to examine for NLG differences between plasma and cell types (CD4^+ ^T cells, CD8^+ ^T cells, PBMC and monocyte) virus *in vivo*. Our data was analysed using the program N-Glycosite [11], available from the Los Alamos National Laboratory HIV Database website [27] (NM, USA). A NLG site is identified in the amino acid sequence by the motif (or "sequon") NX[S/T] with N-Glycosite. The sequon has to begin with an asparagine (N) followed by any amino acid except Proline. The next amino acid residue has to be either a threonine (T) or a serine(S). Both the gap-stripped and gap-inclusive alignments were used in this analysis. Through the gapstripping process, separate regions in the alignment could come together and consequently form an unintentional NX[ST] sequon. This would cause the alignment to register a false NLG site. Through careful examination, we confirmed that no glycosylation sites were accidentally created from the gap stripping process.

### Empirical statistical analysis

The frequency of NLG sites in the sequences found in both plasma and diverse leukocytes (PBMC, CD4^+ ^T cells, CD8^+ ^T cells and monocytes) was examined. The χ^2 ^test was used to compare the frequencies observed across five different compartments for each NLG site identified. A 5 × 2 contingency table was used to evaluate their statistical significance. Next, Fisher's exact test was used to compare the frequencies observed from plasma versus all cell-types together for each NLG site. We further analysed the differences in the mean and median number of NLG sites both across compartments and between patients using the non-parametric Kruskal-Wallis test, as the hypothesis of a normal distribution for the number of glycosylation sites was rejected by the Shapiro-Wilks test. All statistical analyses were performed with the R package [28] (ver. 2.4.1). These statistical analyses allowed us to derive a true snapshot of glycosylation distribution in the 305 HIV-1 *env *gp120 protein sequences from different blood leukocyte populations and plasma *in vivo*.

### Bayesian network analysis

A Bayesian network describes a set of direct dependencies that together explain as much as possible the observed correlations in a dataset [29]. As there could be interdependencies and statistical correlation between individually observed NLG sites, patient grouped sequences and/or compartment categorized sequences, we generated Bayesian networks of all 305 HIV-1 *env *gp120 protein sequences using the procedure developed by Deforche *et al *[30].

For each patient, we created compartmental (PBMC, CD4^+ ^T cells, CD8^+ ^T cells, monocytes and plasma) consensus sequences from our clones. These five consensus sequences for each patient were then used for the Bayesian network analysis. This approach allowed us to remove any false positive associations caused because multiple cloned sequences from the same patient of the same compartment are likely to be highly similar. If the consensus sequences were not used, the arcs of the network could be artificially strengthened and we might overestimate of the significance of some associations. The most probable Bayesian network is the one that maximizes the posterior probability of the model given the data, subject to a prior distribution of model, which we assumed was uniform. We used a simulated annealing heuristic to search in the space of all possible Bayesian network structures [30,31]. To measure the reliability of each of the arcs, a bootstrapping method was used in which 100 replicates of the original dataset were generated by random sampling with replacement, and the most probable Bayesian network re-inferred. Only arcs that occurred in at least 70% of these networks were considered significant for their inclusion in our result.

## Competing interests

The author(s) declare that they have no competing interests.

## Authors' contributions

YSH: Carried out the entire study, designed the framework, developed in-house bioinformatic tools for analysis and wrote the manuscript; ABA, MC, KD, KT and AMV provided highly coordinated help with statistical analysis and Bayesian network analysis; DD provided patients and their enrolment for this study, and provided all the clinical information needed and NKS conceived of the study, and participated in its design and coordination and helped to draft the manuscript. All authors read and approved the final manuscript.
